# Navigating Aged Care Services with GIS: Trends, Developments, and Future Directions

**DOI:** 10.1186/s12877-024-04799-4

**Published:** 2024-03-11

**Authors:** Xuehan Wang, Zhihan Liu

**Affiliations:** School of Public Administration, Central South University, Changsha, Hunan 410004 China

**Keywords:** GIS (Geographic Information System), Application, Aged care services, Scoping review

## Abstract

**Background:**

With the growing challenge of an aging population, emerging technologies are increasingly being integrated into the production, organization, and delivery of aged care services. Geographic Information System (GIS), a computer-based tool for spatial information analysis and processing, has made significant strides in the allocation of care recources and service delivery for older adults, a notably vulnerable group. Despite its growing importance, cross-disciplinary literature reviews on this theme are scare. This scoping review was conducted to encapsulate the advancements and discern the future trajectory of GIS applications in aged care services.

**Methods:**

A comprehensive search across nine databases yielded 5941 articles. Adhering to specific inclusion and exclusion criteria, 61 articles were selected for a detailed analysis.

**Results:**

The 61 articles span from 2003 to 2022, with a notable increase in publications since 2018, comprising 41 articles (67% of the total) published between 2018-2022. Developed countries contributed 66% of the papers, with 45% focusing on accessibility issues. In the domain of aged care services, GIS has been predominantly utilized for model construction, mapping, and site selection, with a growing emphasis on addressing the unique needs of different subgroups of older adults.

**Conclusion:**

The past two decades have seen substantial growth in the application of GIS in aged care services, reflecting its increasing importance in this field. This scoping review not only charts the historical development of GIS applications in aged care services but also underscores the need for innovative research approaches. Future directions should emphasize the integration of GIS with diverse methodologies to address the heterogeneous needs of older adults and improve the overall delivery of aged care services. Such advancements in GIS applications have the potential to significantly enhance the efficiency, accessibility, and quality of care for the aging population.

## Introduction

In the context of burgeoning global economic development, the phenomenon of an aging population is increasingly prominent. It is projected that the world's population will surpass the eight billion mark by November 15, 2022. A noteworthy trend in global demographics is the rise in life expectancy, which reached an average of 72.8 years in 2019, marking an approximate increase of nine years since 1990. This upward trajectory is expected to continue, with projections suggesting an average global longevity of 77.2 years by 2050 [1]. This increase in life expectancy can be attributed to a confluence of factors, including a worldwide decrease in birth rates, advancements in medical technology, and overall societal progress. These factors have not only contributed to extended human life spans but also led to significant shifts in mortality rates and demographic patterns, thereby influencing various facets of global society. In light of these changes, there is a pressing need to focus comprehensively on the healthcare, social security, and emotional well-being of the elderly. This demographic shift calls for a global response, urging nations to address the health requirements of their aging populations. Despite the increase in longevity, the question arises: are the needs for aged care services being adequately met? In response to this critical societal challenge, governments across the world are taking proactive measures. They are formulating and implementing policies aimed at safeguarding the lives of the elderly and fulfilling their diverse needs, thereby ensuring their well-being in the later stages of life.

The spectrum of care services for the elderly encompasses both physical and mental well-being. Ensuring access to comprehensive healthcare services is crucial for maintaining healthy lifestyles among older adults [2]. Care services for this demographic are distinct and more specialized compared to those for younger populations. Given their often-diminished physical robustness, the lifestyle and mental health needs of the elderly differ markedly from those of younger adults. However, there is a notable gap in the existing literature pertaining to care services, with insufficient attention to the multifaceted nature of aged care. A segment of research focuses primarily on the physical health aspects of the elderly, such as oral health, advocating for an expansion in geriatric dental services to meet growing demands [3]. In contrast, other studies emphasize the logistical aspects of accessing aged care services, including the influence of public transport's accessibility and reliability on seniors' ability to reach medical facilities. This diversity of focus highlights the complexity inherent in defining care services for the elderly. Consequently, this paper adopts a broader perspective, encompassing health care, housing, nutrition, and psychological support as integral components of elderly care. Additionally, it addresses factors that directly impact seniors' access to these services, such as the availability of transportation, thereby acknowledging the comprehensive nature of care requirements in older age.

In the realm of aged care services research, the application of Geographic Information Systems (GIS) for analysis and evaluation has garnered attention. Initially proposed by Roger Tomlinson in 1963 [4], GIS technology has undergone significant evolution and found widespread application across various disciplines over the decades. The past fifty years have witnessed remarkable advancements in GIS research, showcasing its potential value in diverse fields and its emergence as a crucial technology in everyday human life. One key area where GIS has shown its utility is in understanding the spatial distribution of medical resources and its correlation with accessibility and availability [5]. The well-being of older adults is closely linked to the availability of healthcare resources, and the spatial distribution of these resources directly influences the distribution of public welfare [6].

The integration of GIS in healthcare has revolutionized the measurement of spatial access to healthcare services [7], enabling more efficient calculation of resource allocation. With advancements in computing technology, GIS techniques have been increasingly used to develop optimal solutions for allocation challenges. These methods are continuously evolving [8–11], aligning more closely with research needs and societal changes. Research that combines socio-economic, demographic, and spatial factors in the context of care services has gained significant interest.

Given the demographic shift towards an aging population, exploring the application of GIS from a technical perspective is pivotal in addressing age-related challenges. GIS can play a crucial role in reducing inequities in access to aged care services. However, the literature on GIS applications within the field of aged care remains comparatively sparse. This scoping review aims to elucidate the specific aspects of aged care services, investigate the precise application of GIS in this domain, and discern the variations across different countries/regions and over time.

## Methods

This scoping review was conducted through a structured five-step process, each step briefly outlined as follows:

### Identification of review questions

Employing the methodological framework established by Arksey and O'Malley, this review delved into the application of GIS within the realm of aged care services. Arksey and O’Malley's seminal work in 2005 laid the groundwork for conducting scoping reviews, providing both rationale and methodological guidance [12]. Adhering to their framework, this scoping review investigated the integration of GIS in aged care services, an area that had seen limited scholarly exploration. By synthesizing three key elements—older adults, care services, and GIS—this paper sought to unpack the intricacies of GIS application in aged care. The review specifically addressed the following three pivotal questions:

1. What are the prevailing demands within aged care services?
2. How is GIS being applied in the sphere of aged care services?
3. Are there any spatial–temporal variations in the application of GIS within this field?

### Literature retrieval

Literature retrieval for this scoping review was conducted across nine databases, encompassing five Chinese databases: CNKI, CSSCI, CSCD, VIP, and Wanfang; and four English databases: Web of Science, PubMed, Elsevier, and EBSCO. A comprehensive search strategy was implemented in these databases without imposing restrictions on publication date or geographic region of the literature. Rigorous documentation and categorization of the retrieved literature were undertaken, laying the groundwork for subsequent screening processes. The specific search strategies employed are outlined in Table 1. The search was completed up to May 2022, hence recent publications from June 2022 onwards were not included. To enhance the efficiency and accuracy of literature deduplication, we utilized the Covidence assessment system software (https://www.covidence.org/), which aided significantly in the preliminary screening of the literature.

**Table 1:** Searching strategies

Database	Keywords	Alternative keywords
CNKI AND Wanfang, VIP, CSCD, CSSCI	GIS	OR “Geographic Information System”
	AND “老年”(older adults)	OR "老年人"
	AND “照护服务”(care service)	OR “健康服务”(health care)
Web of Science AND PubMed, Elsevier, EBSCO	GIS	OR "Geographic Information System"
	Older adult	OR "older adults", "ageing population", "elderly", "geriatric", "older people", "senior"
	Care services	OR "care service", "caring service", "health care"

### Inclusion/exclusion criteria

In order to ensure that the selected literature aligned with our study objectives, an additional research team member was involved in examining the relevance and quality of the papers. Two team members collaboratively conducted the screening of necessary articles. This preliminary screening involved reviewing each article's title, abstract, and keywords to determine its potential inclusion in our study.

The criteria for inclusion in this review were as follows:

1. The primary focus on older adults aged 60 and above.
2. Utilization of GIS-related technology or methodologies.
3. Coverage of care or health services.

Conversely, the exclusion criteria were:

1. Articles that were reviews, conference proceedings, newspapers, books, dissertations, etc.
2. Studies where the target population was not primarily older adults, but included other demographic groups such as children and adults under 60 years old.
3. Articles that did not involve care or health services.
4. Lack of GIS technology or method application.
5. Articles that were not accessible or where the full text was unavailable.
6. Duplicate publications.

### Consultation and determination

During the screening process of articles, instances arose where it was uncertain whether certain literature should be included. In such cases, consultation with another research team member was essential to make determinations based on the established inclusion and exclusion criteria. In scenarios where controversies persisted, a thorough review of the complete text was necessary to ascertain compliance with our inclusion requirements. Despite occasional minor contradictions, these articles were still considered eligible for inclusion in our research.

### Evaluation

To comprehensively evaluate the selected articles, we assessed the overall quality based on five key aspects: research objectives, literature review, methodology, results, and significance. Each aspect of each article was graded according to the criteria outlined in Table 2, ensuring adherence to quality reporting standards [13]. For ease of scoring, we employed a four-level rating system: 0 for not meeting the standard, 1 for nearly meeting the standard, 2 for meeting the standard, and 3 for exceeding the standard. Out of the 65 articles evaluated, scores ranged from 7 to 14. Considering that a score of 2 per aspect indicates meeting the standard, and thus a cumulative score of 10 across all five aspects, articles scoring below 10 were excluded. Consequently, 4 articles scoring under 10 points were omitted, leaving 61 articles that met the criteria for this scoping review and were included in the analysis.

**Table 2:** Quality evaluation

Criterion	Objective	Literature review	Method	Result	Significance
0=does not meet standard	Incomplete	Unable to discuss around the topic; lack of discussion on previous studies	GIS was not applied	Incomplete	Not articulated
1=nearly meets standard	Roughly articulated	Able to engage in discussion within the research field; able to connect with previous work	Applied GIS databases	Basic results; can summarize research findings	Able to articulate but not necessarily practical or persuasive
2=meets standard	Clearly articulated	Clearly explained the current research field; related to previous research; clear expression	In addition to databases, can apply one function of GIS	Complete; able to express results in text and data/charts	Relatively fully articulated
3=exceeds standard	Scientifically and clearly proposed objectives	Able to comprehensively introduce the research field; focusing on the topic; closely linked to previous research; able to propose new personal perspectives	Able to use two or more functions of GIS	Clear expression; able to combine graphics and text for explanation; expand around the topic; able to answer questions raised; able to connect with previous research	Persuasive from theory or practice; unique insights and contributions

### Charting

Accurate and detailed data of the articles meeting the inclusion criteria were meticulously recorded in an Excel spreadsheet. This record included information such as title, author(s), publication year, research area, number of citations, target population, research methodology, and key findings.

## Results

**Fig. 1:**
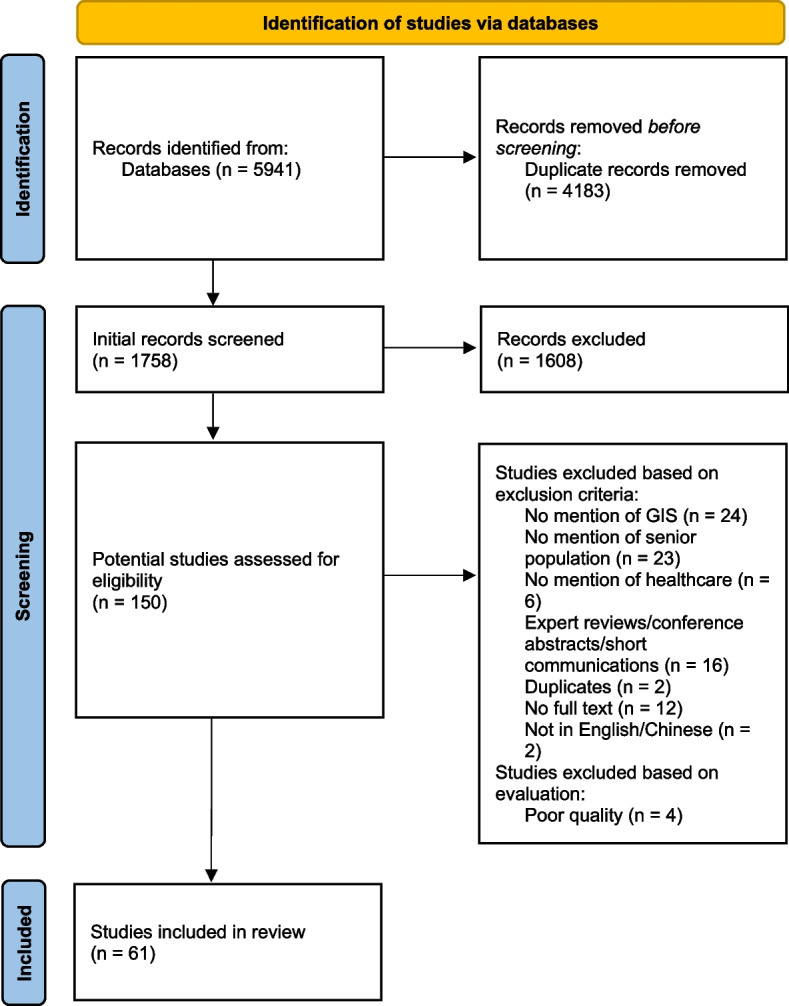
Study selection

Following the aforementioned methodology, we initially screened a total of 5941 articles. Among these, 4183 were identified as duplicates and subsequently removed (as illustrated in Fig. 1). Collaboratively with another team member, we conducted an initial screening of the basic information of the remaining 1758 articles. This process resulted in the exclusion of 1608 articles that did not meet the established criteria. Subsequently, from the pool of 150 potential articles, an additional 89 articles were excluded. Ultimately, 61 articles satisfied all the inclusion criteria and were included in our analysis, as detailed in Table 3.

**Table 3:** Detailed information of included studies

Author & Year	Country/ Region	Characteristics of the older adults	Research Methods	Analyzing methods of GIS	Application	Key findings
Jodi Sturge 2020 [14]	Netherlands	Older adults experiencing memory problems and living at home	Mixed methods: interview, GPS data,	Qualitative GIS	The GPS data, travel diary data and interview transcripts were analyzed using a grounded visualization approach.	A qualitative GIS approach results in an enhanced understanding of activity space patterns.
Jacobo Ruza 2014 [15]	America	Older adults in Palo Alto	Qualitative: case study	Web-based GIS tools	The framework includes assessment using web-based GIS tools.	This study highlighted open spaces, public transportation and services for aged population.
Danny L. Scerpella 2019 [16]	America	250 family caregivers and persons living with dementia	Quantitative: data and trial	GIS analysis, buffers	Geographically predict locations based on data.	This study is a novel usage of GIS methodologies utilized retrospectively to geographically predict locations.
Long Cheng 2019 [17]	China	12,000 households and 35,600 individuals selected in a randomized way	Quantitative: data and model	Spatial expansion model, database	Variables are measured based on the Nanjing city’s GIS database	Older adults have potential access to smaller number of recreational opportunities than younger adults, which may increase elderly’s likelihood of social isolation/exclusion.
Peiman Amini-Behbahani 2020 [18]	Australia	Older adults in residential aged-care centres	Quantitative: clustering method	Calculation	Analyse the walking distance and destinations.	This study highlights the importance of existence of guideline or regulation for the analysis of urban environment prior to allocation of a site or building to residential aged care.
Mark W. Horner 2015[19]	America	People in Leon County aged 65 and up	Quantitative: data and accessibility model	TransCAD’s network analysis and matrix functions	Implementing accessibility models in a GIS environment.	Typically those 85+ had the highest level of accessibility.
Zhu Jin 2018 [20]	America	Adults aged 65 years and older living in northern Manhattan	Quantitative: agent-based simulation model	ABM (agent-based simulation) created by GIS	An agent-based model (ABM) was created with a GIS to simulate the influence of social ties and transportation choices.	Social support may exert substantial influence on the daily activities and health behaviors of older adults.
Tayyab Ikram Shah 2017 [21]	Canada	Senior population (age 65 and over)	Quantitative: model	3SFCA	Examining access to family physician and nurse practitioner services using a GIS-based accessibility approach.	GIS-based accessibility measures are sensitive to the quality of input data and practice considerations.
Zhe Wang 2010 [22]	America	114 older adults from five assisted-living facilities in Houston, TX	Quantitative: multivariate analyses, data analysis	Objective measures (from GIS), GIS data	Characteristics of the environments at the site and neighborhood levels were assessed using GIS measurements.	If the site and neighborhood environments are designed with walking-supportive conditions, older adults may choose to walk more, which in turn can promote their health and reduce the societal demand for senior services.
Qiuyi Zhang 2018 [23]	America	Older adults living in the neighborhoods of northern Manhattan, New York City.	Quantitative: agent-based model, GIS	Data, GIS environment	The spatial dimension is simulated through the travel costs incurred in the GIS-based model environment.	Proximity to screening and treatment facilities is an important factor affecting individual decisions to seek health services.
Tiantian Gu 2018 [24]	China	Elderly people over 60 years old in Nanjing.	Quantitative: two-stage optimization model, greedy algorithm	Data, location specifying	Deep insights into spatial data are revealed by GIS techniques.	The primary data and secondary data are input as the attributes of the point, polyline and polygon. This makes it possible to process as much spatial data as possible through GIS techniques.
Huanhuan Zhu 2021 [25]	China	Elderly aged 65 and above who used the ambulance in 2020	Quantitative: gravity model, empirical Bayesian Kriging (EBK) interpolation analysis	Interpolation, preliminary mapping, buffer analysis, kernel density and overlay analysis	Using GIS-based tools to evaluate the spatial accessibility in conjunction with the spatial distribution of aging people, available road networks, and prehospital EMS facilities.	The elderly’s spatial access to prehospital EMS was imbalanced in the study area.
Yan Ma 2016 [26]	Japan	Elderly in a random sampling in Kanazawa city	Quantitative: ABM	Dataset	The model has been tested by using the real GIS dataset of a Japanese local city.	The agent-based DC center location model could competently simulate the entire Japanese DC center development process.
Zacharias Dermatis 2020 [27]	Greece	A sample of 897 questionnaires, collected from a number of open elderly care centers	Quantitative: data, questionnaires, Spearman test	Data, mapping	Obtained results were presented, using GIS system, as a means to visually illustrate socio-economic indicators in different geographical positions.	The results of the current survey are located automatically in geographic maps as GIS, in order to be shared and analyzed easily.
Elizabeth Hames 2016 [28]	America	1218 older adults over 65 years old in South Florida	Quantitative: principal components analysis (PCA), spatial analysis	Mapping	Create and map age-stratified vulnerability scores using a geographic information system (GIS).	The use of geographic and statistical techniques to explore local factors related to aging demonstrates the benefits of heightened data granularity.
Jean Michael Marcelin 2016 [29]	America	Population age 65 and over in Leon County	Quantitative: p-Median modeling	Data, spatial network optimization analysis	Using ArcGIS 10.2.2 and TransCAD 6.0 to manage the data and to create the final maps for this study.	The Population aged 65 and over when specified as relief demand tends to bear the higher relative burden of the travel time which decreases their overall accessibility to disaster relief.
Yang Cheng 2012 [30]	China	Elderly in Beijing	Quantitative: data and GIS methods	Shortest path analysis, a two-step floating catchment area (2SFCA) method	Two GIS-based methods are used to study the distribution pattern of RCF service areas and spatial accessibility.	The results of spatial accessibility vary by using different methods.
Raj Patel 2019 [31]	Australia	Aging population residing within 50 km of the General Post Office of metropolitan Sydney	Quantitative: data,	Mapping, QGIS	Using GIS to examine the spatial accessibility of the public transport network and dental provider locations.	This knowledge deficit can now be addressed thanks to advances in the field of geospatial analysis, coupled with the decreasing cost and improving usability of GIS
Mark W. Horner 2017 [32]	America	Vulnerable ageing populations	Quantitative: spatial data, vector balancing algorithm	GIS-based network optimisation methodology, spatial data	Operationalising the special needs demand for the shelter modelling was done using GIS. TransCAD GIS3 was used to manage the spatial data.	Outputs of GIS modelling must be weighed against other needs and considerations in the implementation phase.
Terri Lewinson 2016 [33]	America	17 nondriving older adults	Mixed methods: interview, data analysis	Data, mapping, buffering	Using GIS technology to display and analyze community-level data. Public transportation routes were obtained from a County GIS Data Browser.	The ease by which public transit users can transfer buses should be more coordinated, so as to allow for greater access to community resources.
Jong-Hwan Park 2020 [34]	China (Taiwan)	1040 older Taiwanese adults	Quantitative: survey data, binary logistic regression models	Data from GIS	Using data derived from GIS. Neighborhood destinations were assessed using geographic information systems (GIS) software.	Neighborhoods with more utilitarian destinations were associated with excessive sedentary time among older women.
Blake Byron Walker 2019 [35]	Canada	286,211 persons comprising the total population aged 65+ years	Quantitative: Spatial-epidemiological approach, questionnaire, moving-average linear model	Mapping	Each facility location in the study area was mapped using GIS. The calculation of local rates used GIS.	By highlighting regions of high and low service ratios, we were able to identify gaps in access as well as opportunities to improve equity in RC and AL for seniors.
Ke Ruan 2018 [36]	China	People aged 60 and above in each block unit of Xi’an City	Quantitative: evaluation model	Data, potential Model	The urban road distribution data of the research area was established on the ArcGIS platform. The public transportation operation route included in the collection was loaded into ArcGIS.	Solely increasing the projected location of hospitals alone cannot completely solve the unfavorable situation of the low accessibility of HOUHs in cities.
SAMINA Z. IKRAM 2015 [37]	America	Mainly people aged 65 and above	Quantitative: data	Proximity method, 2SFCA	The proximity method uses the distance, and 2SFCA method considers the match ratio between providers and population as well as the complex spatial interaction between them.	Compared to the other groups, African-Americans are disproportionally concentrated in areas closer to their nearest pharmacy in terms of travel time, while white ratios tend to be higher in areas more distant from a pharmacy.
Carlos Mena 2020 [38]	Chile	284 adults with ages from 60 to 74 years old from Talca City	Quantitative: spatial autocorrelation analysis, Moran’s I	Management, processing and analyzing data	GIS analyses were performed to detect global and local geographic clustering.	The distribution of older people registered as frail was found to be associated with certain areas characterized by poor urban infrastructures and socioeconomic levels where high-frailty conditions are commonly present.
Hui-Ching Wu 2018 [39]	China(Taiwan)	3,148,283 elderly individuals (age 65+)	Quantitative: Gini coefficient, “median-mean” skewness, dataset	Model builder, network analyst extension, domain partition OD cost matrix calculation	This study combined sociological perspectives and a GIS-based approach called “domain partition OD cost matrix calculation”.	Community-based care resources can be important social support systems in promoting elderly health.
Nadine Schuurman 2015 [40]	Canada	Elderly aged over 65 years old in Newfoundland	Quantitative: sample phone survey, datasets, gravity model	Spatial model, ODMatrix function, network analyst	Using the ODMatrix function provided within the Network analyst extension in ArcGIS	In each case, a combination of topography, historical settlement patterns and health allocation decision-making have combined to produce unique patterns of access to this end-of-life care.
T. Hanibuchi 2011 [41]	Japan	Japanese elderly aged 65 and over	Quantitative: survey, logistic regression	Spatial analyst	Six types of geographical accessibility to the dental clinics were calculated using GIS.	The importance of the means of transportation as one of the reasons for the gender difference in the geographical access to dental care.
Federica Gaglione 2021 [42]	Italy and England	Aged 65 and above	Quantitative: AHP, multivariate statistical analysis,	Network analysis	The set of variables has been associated in the GIS environment with each arc of the pedestrian graph.	The accessibility levels also identify the critical areas that require priority interventions and the areas where it is possible to increase the levels of pedestrian accessibility to urban services.
Jongjit Rittirong 2016 [43]	Thailand	The elderly (≥60 years) with chronic ailments in Kanchanaburi Province	Quantitative: statistical analysis, data, survey, regression analysis, logit model	Spatial network analysis,	Possible linking travel routes were calculated using GIS spatial network analysis (ArcGIS).	Distance did impact the frequency of healthcare visits, though these effects were weakened when co-residing with an adult child and/or spouse.
Ti-Ching Peng 2020 [44]	China(Taiwan)	Elderly in Taiwan	Quantitative: data, two-stage least squared model (2SLS)	E2SFCA, Spatial quantile regression (SQR) model	With the help of ArcGIS, the addresses were converted into longitudes and latitudes on GIS map layers for spatial measures	Residents living in lower priced neighbourhoods may dislike ambulances’ annoying sound of sirens, while residents living in higher valued neighbourhoods may on the contrary appreciate ambulances’ healthcare services.
Matthew Lee Smith 2018 [45]	America	At-risk older adults in rural areas	Quantitative: data, descriptive statistics	Mapping	Geographic information systems (GIS) geospatially represented the collective reach of the eight interventions.	To increase older adults’ access to and utilization of evidence-based fall prevention programs, interventions must be delivered where older adults feel comfortable and regularly congregate.
Shivangi Prasad 2017 [46]	America	Elderly aged ≥65 years	Quantitative: cumulative distribution functions, multiple comparisons test	proximity analysis, data	Geographic Information Systems (GIS) proximity analysis and cumulative distribution functions were used.	To increase older adults’ access to and utilization of evidence-based fall prevention programs, interventions must be delivered where older adults feel comfortable and regularly congregate.
Chung-Chih Lin 2006 [47]	China (Taiwan)	Elderly persons suffering from dementia	Quantitative: questionnaire, global system for mobile communications (GSM), radio frequency identification (RFID)	GIS parser, image	Convert the longitude and latitude coordinates into a street map location using a GPS and GIS parser.	Analysis of system performance and reliability using different telecoms and different models of mobile phones reveals that the system can provide family members with the patient’s latest location information within 34 seconds.
Takashi Naruse 2017 [48]	Japan	Elderly people living within reach of HVN agencies for each of 17 municipalities in one low-density prefecture	Quantitative: multilevel logistic analysis, data	Locating on the map	The population of elderly people was calculated using public data and geographic information systems.	Municipalities with a higher reachable proportion of elderly residents showed significantly higher HVN (Home visiting nurses) service use rates.
Eoin O’Mahony 2019 [49]	Ireland	People aged 65 years or older to a large hospital in Dublin	Quantitative: retrospective analysis,	QGIS, GIS analysis and visualization, mapping	Address-matched records were analysed using QGIS	Older patients travel shorter distances on average, based on mean straight-line distance from their home address.
Ong Ming Lee Deborah 2018 [50]	Singapore	Singaporeans aged 65 and over	Quantitative: Python, distance decay function	E2SFCA	GIS concept and methods have been utilized to measure geographical accessibility of one major type of primary healthcare services.	This concentration of efforts for a certain demographic group may also mean overlooking the other, namely the elderly.
Gerardo Carpentieri 2020 [51]	Italy	Elderly aged 65 and over in Naples	Quantitative: data, geoprocessing,	2SFCA, network analysis operations, mapping	A geodatabase using GIS software, containing different types of data.	Accessibility to the main urban facilities is clearly a decisive element to guarantee paths for the promotion and protection of wellbeing, in particular for the elderly.
Sunwei Liu 2020 [52]	China	Elderly in 49 communities in Xi’an	Quantitative: mainly GIS analysis	2SFCA, potential model, proximity analysis	The two-step floating catchment area (2SFCA) method and a potential model based on the Geographic Information System (GIS) were used.	Distance is not a determinant factor affecting the elderly to choose community care facilities.
Luisa N. Borrell 2006 [53]	America	Adults aged 65 and older in New York	Quantitative: database	Spatial analysis	ArcGlS software was used to create a GIS incorporating relevant data from a variety of sources.	GIS represents an emerging tool to understand and address oral health and health care disparities among seniors by race/ethnicity and ability to pay for services.
Wisam Kamil 2021 [3]	Australia	People aged 65 years and older	Quantitative: data	GIS analysis	GISs were applied in the analysis of public health-related data.	There is an increasing density of the aged population in socio-economically deprived areas, with inadequate distribution of dental services.
Jung In Kim 2015 [54]	America	Ageing population in Dallas	Qualitative: retrospective case study	Web-based GIS tools	An evidence-based decision-support system, enhanced with a GIS.	GIS tools have the power to display complex analysis results in an easy-to-comprehend, visual manner.
Barbara A. Hirshorn 2003 [55]	America	The 60 and older Lancaster County population	Quantitative: data	Overlay analysis, buffer analysis	The association of attribute and spatial data within a GIS permitted the visualization and analysis of older population-specific needs.	The ability to rapidly update information within GIS permits the analysis of longitudinal spatial processes, making this technology well suited for the evaluation of community intervention effectiveness.
Elena Matei 2018 [56]	Romania	Ageing of the Romanian population	Mixed methods: spatial analysis, in-depth interviews, survey, data	Mapping	Using GIS spatial analysis for mapping the location.	Social support network for the elderly must be continuously adapted to the demands of the society.
Nikolaos Yiannakoulias 2003 [57]	Canada	Community-living residents of Alberta 66 years of age and over	Quantitative: empirical Bayes estimates, Gamma distribution model, Monte Carlo hypothesis	GIS analysis	Using a GIS to describe the pattern of emergency department reported falls of the elderly in the Capital Health Region.	Descriptive geography can enhance the effectiveness of injury prevention programs by identifying zones of high risk.
Yongjiu Xia 2021 [58]	China	Ageing population in Hefei	Quantitative: data	GIS spatial methods, kernel density, network analysis	Geographic coordinates are obtained and imported into GIS. The collected data were visualized by GIS, and a basic database for spatial analysis was established.	Conclusion of evaluation on rationality of spatial allocation of old-age service facilities is closely related to spatial scale.
D. Taylor 2019 [59]	Australia	People who are frail living in Australia aged 65 years or more	Quantitative: data calculation,	Mapping, Geospatial modelling	An example of the data integration capabilities of GIS.	Reducing frailty will lead to benefits in well-being for older Australians in addition to reductions in health care costs.
Tom Carlson 2010 [60]	America	2000 U.S. census data for adults ≥ 65 years of age	Quantitative: data	Proximity analysis, geographic buffers, network analysis	Using GIS to create a site selection strategy.	GIS can be used to determine actual travel time, and may facilitate the selection of community-based prevention program sites to maximize accessibility and utilization by targeted populations.
Min Cheng 2020 [61]	China	Ageing population in Jing’an District	Quantitative: modified immune algorithm (MIA), sensitivity analysis	Spatial optimization, visualization	GIS is used to extract information on spatial relationships and visually display optimization results.	The rational configuration of RCFs helps to improve the ability of urban old-age service.
Kwangyul Choi 2021 [62]	Canada	Ageing population in Calgary	Quantitative: data	Spatial Analysis	Using spatial analysis to identify communities of concern for older adults from the perspective of accessibility.	The application of GIS to perform spatial analyses can be adopted to other areas or regions not only to gain better understanding of the spatial patterns of necessary resources for older adults but also to identify communities of concerns for them.
Long Cheng 2020 [63]	China	Ageing population in Nanjing	Quantitative: data, Gini coefficient	2SFCA, database	A two-step floating catchment area method was utilised to measure accessibility and the Gini coefficient was applied to show inequity.	Upper-tier hospitals are more aggregated and thus more unevenly accessible than the lower-tiers.
Guangping Chen 2020 [64]	China	Older adults aged 60 and over	Quantitative: data, Python, case study, Sensitivity analysis	2SFCA	Based on a road network analysis, a Gaussian two-step floating catchment area (2SFCA) method is employed to calculate EMSA scores.	The elderly in inner-city Wuhan suffer different potential health risk levels at different hours when they must access healthcare facilities.
Rebecca Evans 2016 [65]	Australia	High proportions of over 65-year-old people	Quantitative: data, statistical analysis	Spatial analysis, mapping	A geographic information system was used to assess geographic access.	Tools such as GIS will be increasingly useful for planners involved in health service design at a population level.
Carmen Guida 2021 [66]	Italy	The elderly within the city of Milan affected by Covid	Quantitative: data	Data collection, GIS analysis, visualization, modified 2SFCA, network analysis	A GIS-based procedure was developed to evaluate the elderly’s level of accessibility to primary health services.	Entire neighbourhoods’ elderly populations suffer from very poor accessibility to primary health services and their condition deteriorates even more because of limited services and activities.
Jing Luo 2018 [67]	China	Ageing population in Wuhan	Quantitative: data, sensitivity analysis, Jenks Natural Breaks method	E2SFCA, network analysis	General hospital data included vector GIS format and volume data.	The study on measuring and analysing AMSE may help to understand the seriousness of the ageing problem from the perspective of medical care.
Wendong Chen 2021 [68]	China	Bus services among the older population in Nanjing	Quantitative: mainly GIS analysis	Adjusted Gaussian 2SFCA method,	The Spatial Join tool, ArcGIS, was used to infer the nearest boarding platform.	More health care resources could be allocated to those home stations with low levels of health care accessibility or those which are deemed inaccessible
Sojung Park 2019 [69]	America	Low-income senior	Quantitative: data, principal component analysis (PCA), bivariate analyses (ANOVA)	Network analysis, visualization	Using network analysis, an advanced GIS technique.	Living in subsidized senior housing in disadvantaged neighborhoods may perpetuate socioeconomic disparities in health and well-being for low income older adults.
Yuanhong Ma 2021 [70]	China	Old people aged 60 or above in Harbin	Quantitative: data, spatial analysis	Data, spatial analysis, mapping, weighted overlay method	By clearly identifying gaps for each type of facility, the proposed GIS-NEMA then could be used for identifying the ideal distribution of essential facilities for aging in place in Harbin, China.	China, the GIS-NEMA offers an effective means to identify ideal service networks as Harbin and other cities in China build age-friendly cities.
Federica Gaglione 2019 [71]	Italy	People aged 65 and older	Quantitative: data, spatial analysis	Data, spatial analysis, mapping	For the environmental, physical and functional subsystems quantitative data are obtained through spatial analysis in the GIS environment.	Integrated actions should be taken both on the functional and physical subsystems, in order to improve urban accessibility and guarantee social inclusion for the elderly by enabling them to actively participate in ‘urban life’.
Yanyan Gao 2022 [72]	China	The elderly, aged 65 and above, treated in medical facilities in Hefei	Quantitative: questionnaire, location-allocation models, network analysis	Network analysis, mapping	The optimization scheme for the layout of medical facilities could be obtained by constructing a location–allocation model in ArcGIS.	The actual needs of the elderly should be taken into account when discussing their issues.
Hyemin Cho 2021 [73]	South Korea	Elderly people in South Korea	Quantitative: data	Network analysis, spatial analysis, mapping	Generating a map that includes road networks, public transportation routes and stops, and welfare facilities using a geographic information system (GIS).	To improve the accessibility of low-accessibility areas, it is suggested that modifying the existing bus routes or adding new transit lines would reduce travel time of the elderly to welfare facilities.

### Characteristics of included studies

The linguistic composition of the 61 articles included in our analysis was predominantly English, with 60 articles written in English and only one article in Chinese. Geographically, the distribution of research areas was varied: 24 articles originated from Asia, including China, Thailand, Japan, Singapore, and South Korea; 23 articles were from North America, specifically Canada and the United States; Europe contributed 8 articles, encompassing Greece, Italy, the Netherlands, Britain, and Ireland; Oceania, represented solely by Australia, accounted for 5 articles; and South America had one article from Chile, as detailed in Table 4.

**Table 4:** 61 Articles distributed by continent and country

Continent	Country	Number	Sum
North America	America	18	23
	Canada	5	
South America	Chile	1	1
Asia	China	18	24
	Japan	3	
	Singapore	1	
	South Korea	1	
	Thailand	1	
Europe	Greece	1	8
	Ireland	1	
	Italy	3	
	England	1^a^	
	Netherlands	1	
	Romania	1	
Oceania	Australia	5	5
Total		61	61

^a^As the research area of article [42] spans both Italy and England, for statistical convenience, this article has been categorized under “England”

Regarding the publication timeline, all articles were published in the twenty-first century, ranging from the earliest in 2003 to the most recent in 2022. Within the Asian context, 18 out of 24 articles focused on China, with 17 of these emanating from China’s first-tier cities or provincial capitals such as Beijing, Shanghai, and Nanjing. Taiwan was the subject of four articles, and the remaining article focused on a non-provincial capital city. These findings indicate that aged care service resources and the application of related technology tend to be concentrated in economically prosperous urban areas, resulting in a greater diversity of research outputs.

**Fig. 2:**
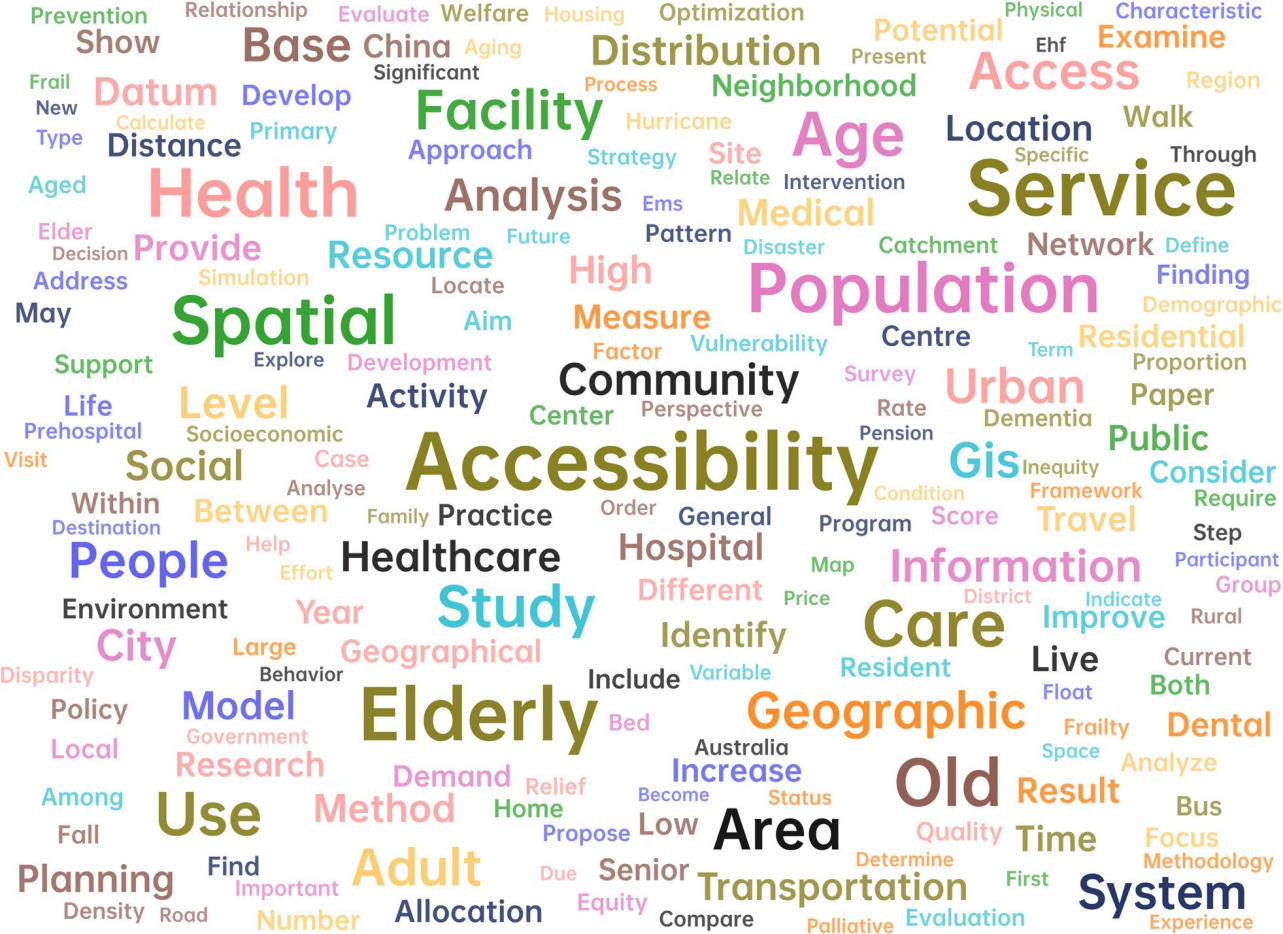
Word clouds of 61 articles

**Fig. 3:**
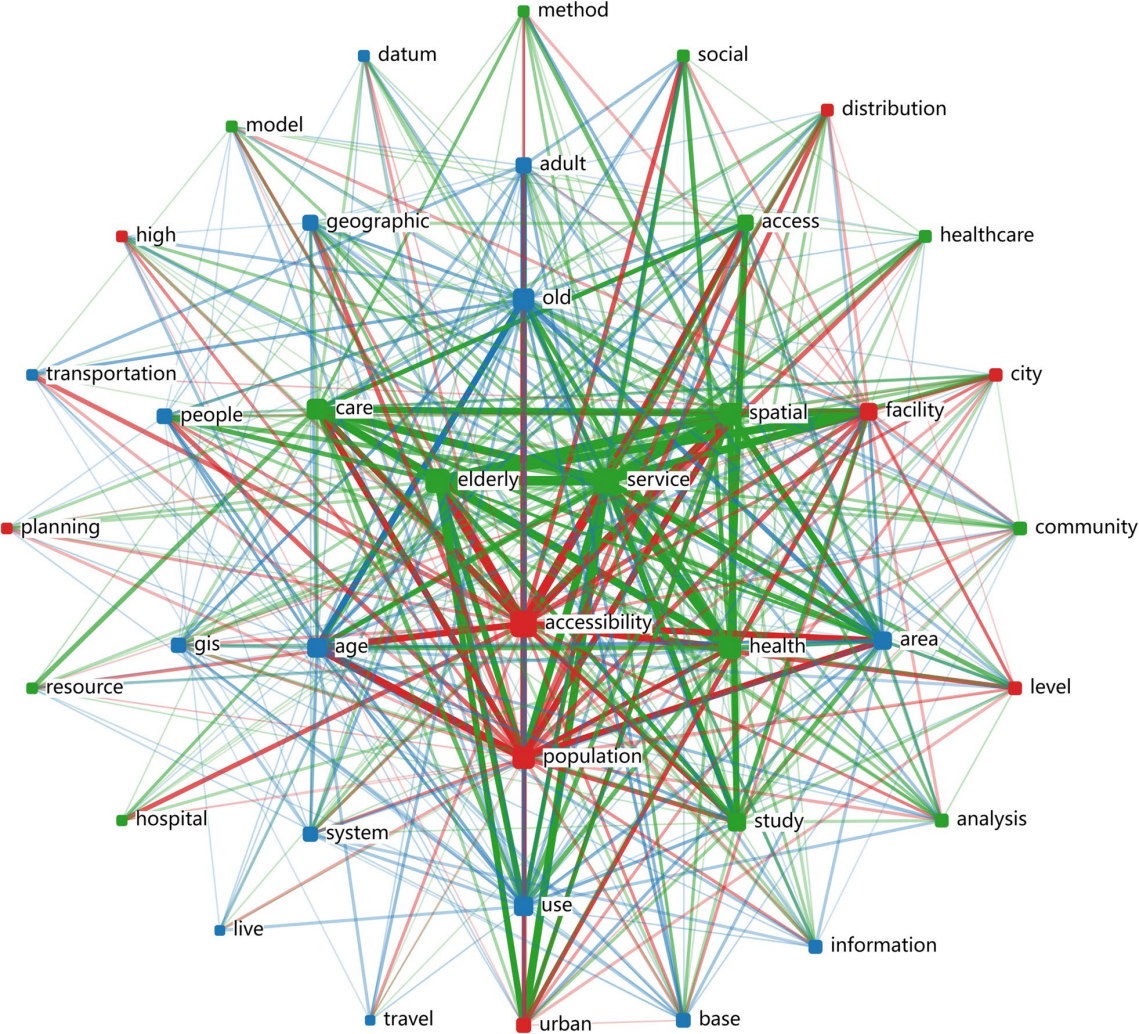
Connection diagram

Utilizing the titles and abstracts of the 61 articles, word clouds and connection diagram were generated after excluding irrelevant words, such as prepositions and adverbs. These visualizations are depicted in Fig. 2, 3. As evident from Fig. 2, the term 'accessibility' emerges with the highest frequency, closely followed by 'elderly', 'service', and 'spatial'. Figure 3 illustrates robust connections between keywords like 'elderly', 'service', 'accessibility', and 'population'. This visualization underscores a prevalent research focus on accessibility and the strong association between older adults and the services they require. These figures not only reflect the thematic emphasis within the existing literature but also highlight the interconnected nature of these key concepts in aged care services research.

### Quality of articles included

Based on the Journal Citation Reports (JCR) data from 2021, the quality of the articles included in this review can be assessed. Out of the 61 articles, 28 were published in Q1 journals, representing approximately 46.0% of the total. Meanwhile, 29 articles were published in Q2-Q4 journals, accounting for about 44.3% of the selection. However, for 4 articles, the journal quality could not be determined due to either unavailability of information or inconsistencies in judging standards across different countries. Overall, the collected articles can be characterized as of moderately high quality.

### Characteristics of target population

The target population in the 61 articles under review was primarily adults aged over 60, although the specific demographic varied across studies. 16 articles focused on older adults within a specific age range, whereas the remaining 45 did not differentiate among senior groups. Specific subpopulations were the focus of some studies: 3 articles investigated older adults with dementia [14, 16, 47]; 4 articles centered on vulnerable elderly individuals [28, 32, 38, 59]. The subjects of the remaining 9 studies included older adults with disabilities [48], those unable to drive [33], individuals with chronic diseases [43], high-risk seniors [45], elderly prone to falls [57], those affected by COVID-19 [66], low-income older adults [69], and those in need of public transportation services [68].

### Care services and demands of older adults

Aging is often associated with a decline in physical function, which in turn leads to changes in needs and demands. Among the 61 articles reviewed, the needs of older adults were categorized into medical care, basic living, transportation, and spiritual needs. A total of 37 articles focused on medical care [3, 15, 16, 20, 21, 23, 24, 28, 29, 31, 32, 36, 37, 39–41, 43, 45–50, 50, 51, 53, 54, 56, 57, 60, 63–68, 72], 14 articles addressed basic living needs [14, 18, 26, 27, 30, 35, 38, 44, 52, 55, 58, 61, 62, 69], 8 articles discussed transportation needs [19, 22, 25, 34, 42, 70, 71, 73], and 2 articles explored spiritual needs [17, 33].

These needs encompass various care services for older adults. Medical care needs primarily included oral health care [3, 20, 31, 41, 53], family physician and nurse practitioner services [21], medical and health facilities and services [24, 36, 37, 49, 50, 54, 63, 64, 66–68, 72], disaster relief [29, 32, 46], palliative care [40], primary health care [51, 65], and fall prevention [57, 60]. Basic living needs covered areas such as aged care facilities or services [18, 30, 52, 58, 61], daily care services [26], housing [44], etc. Transportation needs involved daily activities [19, 34] and walking [22, 42]. Spiritual needs comprised recreational facilities [17] and religious beliefs [33].

### Application of GIS

In the reviewed articles, the application of GIS was demonstrated through various methodologies. Among the 61 articles, the 2SFCA (two-step floating catchment area) method was employed in 11 articles [30, 37, 44, 50–52, 63, 64, 66–68]. Ten articles utilized ArcGIS software [29, 36, 39, 40, 43, 44, 61, 62, 65, 68], and another ten articles applied the database functions of GIS [3, 17, 38, 48, 51, 53, 55, 58, 63, 67]. Six articles combined GIS with case study approaches [15, 50, 51, 54, 63, 64], while another six focused on spatial analysis [28, 43, 53, 55, 56, 62]. The ABM (Agent-Based Modeling) method was used in three articles [20, 23, 26]. Notably, 27 articles employed GIS to study accessibility issues [17–21, 23, 25, 29–31, 33, 36, 37, 39–42, 44, 46, 48, 50–52, 63, 67–69].

### Spatial–temporal differences in application

From a historical perspective, the earliest studies included in this review were published in the United States and Canada in 2003, while the most recent publication originated from China in 2022. The temporal distribution of the articles shows a marked increase in recent years: a total of 20 articles were published from 2003 to 2017, and a significant uptick occurred from 2018 onwards, with 41 articles being published in the span from 2018 to 2022.

Geographically, the majority of research output originated from Asia and North America, accounting for a combined total of 47 articles, which is approximately 77% of the total. Within Asia, China was the most prolific, contributing 18 articles. Most of these Chinese studies were focused on the distribution or accessibility of medical service resources and community aged care services, displaying a relative consistency in research topics. In contrast, the 23 North American articles, with 18 of them from the United States, showcased a diversity of research topics, often centering on case studies in various regions.

## Discussion

The analysis of the 61 articles in this review underscores that GIS applications exhibit varying characteristics and play diverse potential roles in aged care services, catering to different populations and service needs. Over nearly two decades, from 2003 to 2022, the utilization of GIS in aged care services has evolved considerably, particularly in the last five years, where development has been notably rapid. However, there still exist limitations in research methodologies and the specificity of target populations. This scoping review, conducted after a thorough screening and retrieval process, represents the first of its kind to focus on GIS applications in the field of aged care services.

The included articles showcased a variety of methods for applying GIS. Common approaches encompassed GIS-based modeling, creating illustrations with GIS, positioning and site selection, and utilizing a GIS database. Firstly, several articles integrated GIS with models, predominantly employing ABM and 2SFCA methods, accounting for 14 articles in total. ABM facilitated nuanced simulation and prediction, allowing for the construction of 'hypothetical' scenarios from multiple perspectives. This approach proved versatile in addressing diverse problems, meeting the needs of various groups, and aiding decision-making processes. The 2SFCA method was primarily used to assess accessibility and relative ease of access to different aged care service resources. Secondly, generating images using GIS technologies, particularly through software like ArcGIS, was a widely used function. For instance, researchers utilized this capability to map feasible routes for older adults to access services, enhancing the efficiency of public resource utilization and catering to the needs of vulnerable elderly populations [33]. This application aids in comprehending the distribution of facilities, mitigating resource distribution inequality, and visualizing care services. Furthermore, GIS was employed for location-specific purposes, such as identifying new sites for specialized medical shelters [32]. Finally, some studies leveraged the information storage and management capabilities of GIS to gather data on public infrastructure locations and images, or to select sites for public facilities.

To accurately address research objectives, researchers often opted for multiple methods rather than relying on a singular approach. This multipronged strategy enabled a more nuanced analysis suitable for the complexities of each study. For instance, one study utilized GIS software to create a geographic database and developed a GIS-based procedure to evaluate accessibility levels of urban services for the elderly. It selected the local primary health service in Naples as a case study and employed the 2SFCA method for evaluation, revealing significantly low levels of community accessibility [51]. Such complex problems often require a multifaceted methodological approach to yield scientifically sound and reasonable results. Overall, GIS applications were diverse, with their specific uses varying both spatially and temporally, thereby enhancing their utility in research.

From 2003 to 2022, methods in aged care service research evolved from singular to more complex approaches. Initially, researchers focused on building theoretical frameworks, gradually incorporating various models and application software to translate theory into practical, applicable activities. The evolution in the aged population research mirrored this diversification. The target demographic shifted from a general, homogeneous elderly population to more specific, heterogeneous groups, including frail, chronically ill, and low-income older adults.

For instance, a Canadian study in 2003 utilized GIS to analyze fall patterns among older adults based on emergency department reports in specific regions, employing the empirical Bayesian method for prediction in Edmonton [57]. This approach, though relatively straightforward, combined qualitative GIS methods with statistical analysis to draw universally applicable conclusions, setting a precedent for subsequent research. However, it lacked consideration of personal, environmental, and social factors. In contrast, a 2011 study from the United States advanced this research by using GIS to assist health planners in developing site selection strategies for fall prevention among older adults, employing ArcGIS’s Network Analyst Extension [60]. This study improved upon earlier research by considering geographical distribution, mobility, and actual travel time to destinations for older adults, thus encompassing more comprehensive factors. However, it did not account for the technical capacity of public health planners or changes in population density.

The distribution of research publications reveals that developed countries accounted for 40 papers, surpassing the number from developing countries, with a more diverse range of application methods. Developing countries contributed 21 papers, 18 of which originated from China, with only 3 from other countries. This disparity underscores the influence of economic development on scientific research capabilities and the extent of attention researchers devote to social issues. Nations with a higher volume of publications tended to explore more varied topics and focus on different demographic groups. For instance, the significance of oral care is well-recognized, and the dental industry in developed countries is well-established. All five articles on oral health care were from developed countries. Additionally, transportation accessibility is a crucial aspect of health resource accessibility, impacting the efficiency, effectiveness, and level of care resource access for older adults. Of the 27 articles focusing on accessibility, those from countries other than China were predominantly from developed nations like the United States, Japan, and Australia. It is also important to acknowledge that the needs for care services among older adults vary across different cultural backgrounds, and public facilities, the social environment, and local policies exert varying degrees of influence on aged care [74]. Future research should consider socio-economic factors more thoroughly.

GIS, with its use of image data and calculation results, offers an intuitive understanding of the relationships between people and land, and between different land areas. Spatial analysis in GIS underscores the value of potential service spatiotemporal acquisition patterns. Data plays a vital role in GIS applications, especially when employing certain models and methods, where it relies heavily on data support. In studying the impact of public care services on older adults, the use of public data is essential. However, the transparency of public data is limited, and not all necessary data are readily accessible. Therefore, relevant public sectors and institutions could enhance data openness, establish public databases, and ease the challenges of data acquisition, enabling more effective research. Future developments might see GIS applications tailored for different institutions or departments, with research and development personnel creating features specific to various institutional types. For users, the operating interface and functions should be as user-friendly as possible.

Beyond data release, ethical considerations regarding data are also paramount. Although GIS databases typically do not involve highly specific personal data like home addresses or phone numbers, it is crucial for relevant sectors and institutions to prioritize the privacy and data security of older adults, implementing multi-level data management. If feasible, the formulation of laws and regulations in this regard might be beneficial.

Additionally, it is observed that resources are often concentrated in densely populated urban areas. Within the 61 articles analyzed, 18 were focused on China, with 17 of these studies based in either first-tier or provincial capital cities. This trend indicates a tendency for researchers to focus on regions with more abundant resources. Large cities, with their dense populations and attractiveness to younger demographics, exhibit a certain balance in their population structure. In contrast, smaller cities often grapple with the migration of younger individuals to larger urban areas, exacerbating the aging issue and receiving less research attention. Consequently, there is a need for future research to focus on these less prominent cities and the aged care services they require.

An examination of the Web of Science database for reviews linking GIS and care services yielded 14 results, but none specifically targeted the elderly. This gap highlights the significant research value and importance of our work. Nonetheless, our review has its limitations. Firstly, due to the constraints in available literature, it includes only those articles retrieved before May 2022. Secondly, while this paper provides an overview of the specific GIS analysis methods used in the included literature, especially the more pertinent models, a more detailed discussion of these methods is warranted in future research. Given the universality of aging, this review holds both theoretical and practical significance.

As the demographic shift towards an aging population accelerates, urban planning must adapt accordingly, particularly in developing countries where rapid urbanization is significantly altering people's lives. Enhancing the city’s environment to be more elderly-friendly requires bolstering supportive measures, both physical and non-physical, to promote the sustainability of health care and social services. Given that GIS applications frequently involve data on population, economy, or facilities, governments could benefit from dynamically and timely updating relevant databases to aid in decision-making. The precision of data directly correlates with the realism of the results obtained.

Different countries should formulate and enact laws and policies that reflect their unique characteristics. In these policies, especially those concerning the allocation of aged care services or the planning of future resources, GIS can be a valuable tool for implementation. Considerations of efficiency and fairness are crucial in these allocations. For instance, in China, it is important that central and local policies align, are feasible, and effectively implemented. Moreover, problem analysis between different provinces and cities should be grounded in their specific realities and circumstances.

Future research should delve deeper into the care service needs of distinct subgroups within the elderly population. The characteristics of these groups can vary significantly, necessitating focused attention on vulnerable segments, such as individuals suffering from Alzheimer's disease, whose needs and challenges differ markedly from those without the condition [14].

The selected articles indicate nascent studies on the application of GIS to address the needs of older adults during the COVID-19 pandemic. Future investigations into COVID-19-related issues should pay closer attention to the unique care service requirements of older adults, encompassing both material and spiritual needs. GIS methods and technologies enable spatial and temporal interaction, which is particularly relevant as the needs of older individuals evolve throughout the course of the pandemic. Understanding the changing needs of different age groups at various stages will be a critical area of focus. To address the issues of inequitable distribution and inadequate accessibility of care services, it is advisable to integrate GIS with simulation methods such as ABM. This approach could enhance the delivery and effectiveness of care services to older adults and other vulnerable populations, ensuring their needs are met more comprehensively and effectively.

## Conclusion

As the inaugural scoping review focusing on the application of GIS in aged care services, this study has illuminated the considerable development and maturation of GIS technology and its applications over the past two decades. GIS offers valuable insights by leveraging geographic information systems and spatial analysis, thereby underscoring the importance of potential spatio-temporal acquisition patterns in aged care services. This, in turn, enhances efficiency and fairness in service distribution. The application of GIS in this field exhibits distinct spatial and temporal variations. Developed countries have been early adopters of GIS, enabling them to address the needs of specific subpopulations of older adults more effectively. In contrast, research in developing countries has been relatively limited, with a notable lack of diversity in research methodologies.

The diverse needs of older adults have consistently been a focal point of research, leading to continuous enhancement in the understanding and provision of care services. Researchers from various countries and regions are increasingly focusing on localized research, enriching and completing the application of GIS in this field. Theoretically, there is a need for innovative methods and techniques, with research approaches being adaptively tailored to the specific issues under study. Practically, the varying needs of different age groups within the elderly population must be considered, and relevant public sectors and institutions should take responsibility for ensuring the efficiency and fairness of care service allocation. Moreover, data is a crucial component of GIS, and effective analysis relies on the availability of accurate and moderately open data sources. Moving forward, attention must be paid to the accessibility and precision of data to further the efficacy and impact of GIS applications in aged care services.

## Data Availability

The present study is exclusively based on data extracted from previously published research. As such, this scoping review does not involve any original primary data collection. All data utilized and analyzed in this study are derived from the original papers referenced herein. Consequently, there are no separate datasets to share beyond what is already available within the articles cited in this study. Readers can find the data supporting the findings of our research comprehensively detailed within the body of this article.
